# Revealing faces: Gender and cultural differences in facial prominence of selfies

**DOI:** 10.1371/journal.pone.0205893

**Published:** 2018-10-31

**Authors:** Nenad Čuš Babič, Tadevž Ropert, Bojan Musil

**Affiliations:** University of Maribor, Faculty of Arts, Koroška cesta, Maribor, Slovenia; California State University Monterey Bay, UNITED STATES

## Abstract

An international sample of 2754 selfies uploaded to Instagram that form part of the Selfiecity (www.selfiecity.net) research project (selfies originating from Bangkok, Berlin, London, Moscow, New York, and Sao Paolo) were examined to assess the existence of facial prominence differences in depictions of males and females and the variability of facial prominence among cultures. Results show that gender stereotypical bias resulting in greater facial prominence in depictions of men is present in selfies. The control of image creation and selection for publication by the persons presented in the images do not diminish this gender based bias. Also, when gender is controlled, significant differences exist in facial prominence among different cultures. Comparisons with various socio-cultural indicators indicate possible correlations of gender stereotypical bias to self-expression values, freedom of choice, people’s influence on government’s decisions, protection of freedom of speech and people’s influence on issues in the professional and communal environment. This research does not find a correlation of gender based bias in selfies with gender equality or inequality measures among cultures.

## Introduction

The selfie, defined as, “a self-portrait photograph of oneself (or of oneself and other people), taken with a camera or a camera phone held at arm's length or pointed at a mirror, which is usually shared through social media” [1] has become a widespread form of self-presentation in recent years. According to a survey from February 2014 Pew Research [2] 26% of all adult U.S. citizens had shared a selfie on a social networking site, while 55% of people aged 18–33 had shared a selfie. Selfies differ in a variety of ways; previous studies address distinct aspects, such as cheek side prominence [3,4], photo filter usage [5], or the number of people in the photo [1] or address the holistic coding schemes of selfies [6,7].

One previously unstudied, but potentially meaningful aspect of selfies is their variation in facial prominence, i.e. the ratio of the head to the total visible body including the head in the depiction of a person. Facial prominence in selfies is relevant because the degree of facial prominence has a variety of consequences for social perception. Studies have consistently shown that persons depicted with higher facial prominence are perceived as more dominant [8–10], ambitious [8,11,12] and assertive [10,11] than persons with lower facial prominence. Evidence for higher facial prominence affecting the perception of intelligence is supported by Archer and Schwartz [11,12] and not supported by Zuckerman [8,9] and hence inconclusive. Also, most studies found no significant effects on positive general evaluation [8,10,11], with the exception of Schwarz [12], who found that higher facial prominence affects positive general evaluation, but only for female perceivers.

This study explores how gender related bias in the facial prominence in selfies appears in different cultures. Additionally, it compares the differences in the facial prominence in selfies collected from different cultural backgrounds.

## Gender differences in facial prominence

Studying facial prominence in the context of gender, Archer [11] coined the term face-ism, defined as “greater facial prominence in depiction of men” (p. 725). Initial research on face-ism focused on mass media depictions of gender [13,14], i.e. in media where editors and professional photographers are the ones who decide about the details of photographs, like head-to-body ratio. In recent years, research has shown that even in mainstream magazines where face-ism used to exist widely, it has reduced but not completely disappeared [14,15]. Nor is it universal across all media or in any particular genre; for example, some magazines display no decline in face-ism over time (e.g., *Newsweek* from 1985 to 2005), while in others face-ism has declined heavily (e.g., *Times*) [15].

More recently, together with the widespread use of smartphones with cameras, extensive taking of self-portrait photographs has become a global phenomenon. In addition, since the advent of social media, everyone is the (potential) editor and publisher of their own photographs. Some popular social networking sites are primarily used for photo-sharing, such as Instagram, which is used by 28% of adult internet users, 31% of women and 24% of men [16]. Thus, control over the final look of portrait photographs, at least on social media, has shifted from third person professional authors and editors towards the first-person authors of the selfie.

But does this shift in control lead to a disappearance of face-ism? Analyses of self-selected profile photos on social networking sites [17,18] have shown the opposite: that self-selection reinforces gender stereotypes. A study of photos of German university professors and politicians, who could presumably self-select their photos, also showed significantly higher facial prominence of men [19]. More importantly, recent content analysis of selfies has shown that they are even more gender-stereotypical than traditional media photographs [20]. It seems that selfies and the self-selection of images posted on social network sites reinforce gender related stereotypes instead of deflating inequalities. In relation to face-ism, we formulated the first hypothesis as follows:

H1: *Selfies posted on the social network platform Instagram display greater facial prominence in depiction of men*.

### Cultural differences in face-ism

Two separate questions arise when analysing the connection of culture with face-ism: whether face-ism is a cross-culturally universal phenomenon, and whether differences in the face-ism index occur between cultures.

Before we address these two questions, we would like to point out that in the literature of contextualised comparative analysis there is often a confusion between assessment of countries, nationalities and cultures. For example, Matthes, Prieler and Adam [21] in the analysis of gender-role portrayals in television advertising across the globe used country and culture interchangeably. The authority of cross-cultural studies Hofstede [22] pointed out that despite nations or national studies (and also consequently countries) are not the best units for studying cultures, they are usually the only kind of units available in comparative contextual research. Therefore, in this research we are also using country and culture interchangeably.

#### Universality of face-ism

The first cross-cultural study of face-ism was part of a seminal paper on face-ism [11], which found face-ism bias in periodicals from all the 11 countries or regions included in the study (Chile, England, France, Federal Republic of Germany, Hong Kong, India, Italy, Kenya, Mexico, Middle East, Spain and U.S.A.). Based on this evidence, Archer and colleagues [11] proposed that face-ism is found across different cultures, i.e. that it is universal. Other studies reported that differences were not significant [14,23]. In an information technology context, Prieler [24] compared the face-ism index on online dating sites in South Korea, Japan, Sweden and the United States and found that face-ism was present in only South Korea and Japan. Konrath, Au and Ramsey [25] investigated face-ism in 25 countries based on photographs of politicians found on official web sites. Face-ism bias was identified in only 15 countries, and 3 countries showed higher face-ism index for women [25]. The same research yielded the paradoxical finding that face-ism was more pronounced in more gender equal cultures [25].

These mixed results in other media stimulated us to check Archer’s assumption in the context of selfies. Hence, we formulated the second hypothesis as follows:

H2: *Face-ism bias is culturally universal in selfies posted on the social network platform Instagram*.

#### Cultural differences in face-ism index scores

Besides face-ism as a phenomenon related to differences in display influenced by the gender of the person presented, intercultural research reveals one more aspect of facial prominence. Facial prominence is measured by face-ism index, which is the ratio of the distance from the top of the head to the bottom of the chin, divided by the distance from the top of the head to the lowest visible part of the body. In comparing results from various countries, the face-ism index varies from country to country [11,18,25].

However, previous research on this topic is scarce. Smith and Cooley [18] tried to explain these differences using Hofstede’s dimensions, but researchers in general agree that Hofstede’s masculinity index cannot explain face-ism [24]. Research on stereotypical depiction of men and women in television advertisements from Asian, American and European countries shows that gender stereotypes can be found around the globe and that comparative studies involving different countries are needed [21]. Additionally, advertising research shows that even in the same country advertisements targeting people from different cultural backgrounds depict human bodies consistent to the cultural differences in gender role expectations [26]. Also, a comparison of Facebook profile pictures between East Asian and American Facebook users shows marked cultural differences in context-inclusive styles versus object-focused styles [23].Because the differences are present in other media or other types of online presentation, we formulated the third hypothesis as follows:

H3: *The face-ism index in selfies posted on the social network platform Instagram varies with the country of origin of the selfies*.

#### Socio-cultural indicators

Since previous research was not productive in the search for an explanation of intercultural differences, we explored two novel paths. We compared the face-ism effect size among countries from our sample with several indices derived from the World Value Survey (WVS) [27]. The survey studies human beliefs related to economic development, the progress of democratic institutions, the rise of gender equality and the efficacy of government. It was conducted in more than 90 countries with a sample average of 1200 respondents per country, covering more than 90% of the world population. Inglehart and Welzel claim that there are two major dimensions of cross cultural variation in the world, and they derived two major indices from WVS variables to score the countries [28]. The first dimension is Traditional values versus Secular-rational values. Secular values dissociate people from external sources of authority. The second dimension indicates emancipative values, which are Survival values versus Self-expression values, where Self-expression indicates freedom of choice and equality of opportunities. In our research, we assumed that the emancipative values would be particularly salient in expressing the potential for explanation of facial prominence differences, since the components reflect tolerance for foreigners, variety in sexual orientation and gender equality.

Additionally, we checked how face-ism effect size might relate to the human development index (HDI) and gender inequality measures (GII), as measured by the United Nations Development Programme [29]. The programme understands development in terms of the richness of human lives and not sole by economic wealth. The GII reflects how women are disadvantaged in empowerment, economic status and health.

Due to explorative nature of this part of the research, we have not formulated any hypothesis. Instead, we have tried to answer a research question:

*RQ1*: *Are there any relationships between gender related facial prominence bias in selfies and socio-cultural measures from WVS and UNDP*?

## Method

### Sampling

We used data from the Selfiecity research project, where all the selfies are publicly available on the project’s website www.selfiecity.net [30]. Initially, they collected 808,000 photos from the photo sharing social network site Instagram, involving six cities around the world (Bangkok, Berlin, London, Moscow, New York and Sao Paolo). The selfies of users from Bangkok, Berlin, Moscow, New York and Sao Paolo were collected from December 5 through December 11 2013, while the selfies of users from London were collected from September 21 through September 27 2015. During various stages of their sampling process (for details see [30]) they recruited Amazon Mechanical Turk workers to examine and exclude any photos that were not selfies. Researchers then re-examined the selected photos and excluded additional errors. The final set contained 640 photos for each city, 3840 photos altogether. We analysed this final dataset, and after applying selection criteria (see below), our total sample (N = 2754) included 1944 female selfies (71%) and 810 male selfies (29%). Authors of the Sefiecity project fully complied with the terms of service of the website Instagram from where they collected the images.

### Measures

#### Face-ism index

Facial prominence is operationalized as face-ism index–a ratio of two measurements [11]: (1) the numerator is the distance from the top of the head to the lowest point of the chin and (2) the denominator is the distance from the top of the head to the lowest visible part of the body in the photo. The index can range from 0, when there is no face depicted, to 1, when only the face is depicted without any other part of the body. The face-ism index was measured with ImageJ 1.50e software (National Institute of Health, USA).

When the body axis of the person depicted in the photo was tilted, prior to measurement the photo was rotated until the body axis was at right angles to the horizontal axis. Following Archer et al. [11], in cases when parts of the head were masked by clothing (e.g., with a scarf, cap or hood), hairstyle, beard or hands, location of the top or bottom of the head was estimated by the coder.

#### Gender

The gender of a person depicted in the photo was determined based on gender-related visual attributes of the face. A subset of the photos was coded by the two coders and there was no disagreement between the coders.

#### Sociocultural measures

Measures tested for correlation with differences in face-ism index scores and face-ism effect size among countries were taken from the two sources, namely WVS and UNDP. From both sources, results from the last available wave at the time of this research (year 2014) were used in this study:

Indices derived from WVS variables [27]:

**SACSECVAL**: this indicates overall secular values ranging from 0 (the most sacred values), to 1 (the most secular values); variable referring to devoutness to the parents, respect for authority, and national pride, the importance of religion, religious practice, and the respondents’ self-perception as religious or not, the relativism towards cheating and bribe, and scepticism toward armed forces, police and the court.

**RESEMAVAL**: this indicates emancipative values ranging from 0 (obedient values), to 1 (emancipative values); it is calculated from 4 sub-indices (described below): AUTONOMY, QUALITY, CHOICE and VOICE.

**AUTONOMY:** indicates attitudes toward independence, imagination and obedience as important values for children.

**EQUALITY:** indicates attitudes toward equality of women and men with regard to jobs, the importance of education and political leadership.

**CHOICE:** indicates whether homosexuality, abortion and divorce could be justified.

**VOICE:** indicates the top priorities of the country with regard to aims such as people’s influence on government decisions, protection of freedom of speech and people’s influence on issues in the professional and communal environment.

All those sub-indices range from 0 to 1. For the theoretical basis and further details about the construction of indices and the corresponding methodology, see [28].

Indices taken from the UNPD survey [29]:

**HDI**: the human development index, reflects the average achievements of countries in three basic aspects of human development, which are as follows: leading a long and healthy life (measured by life expectancy at birth); being knowledgeable (measured by mean years of schooling) and enjoying a decent standard of living (measured by gross national income per capita). HDI reflects the results of a country’s national policy choices about human development.

**GII:** the gender inequality index, measures gender inequalities in reproductive health (measured by the maternal mortality ratio and adolescent birth rates), empowerment (measured by the involvement of females in government and female education levels), and economic status (measured by the labour force participation rate). GII shows the gender gaps in major areas of human development.

Both indices range from 0 to 1. For details about the calculation of HDI and GII, see Technical notes in the UNDP report [29].

#### Selection criteria

Photos were selected using the following criteria based on Archer’s guidelines [11] (Selection criteria in italics are the original Archer’s criteria. Our additional criteria, which are adjusted to specifics of selfies are printed in normal font.):

1. *Only one person is depicted in the photo*.
2. The photo is a selfie, i.e. it was taken by the person in the photo.
3. The photo is not blurred.
4. *The photo contains no “co-subjects” (e*.*g*. *animals*, *objects or landmarks) or hand gestures*.
5. *When there were multiple photos of the same person*, *only the first photo in the dataset was selected; others were excluded*.
6. The gender of the person depicted was unambiguous. Whenever gender was ambiguous, the given photo was excluded.
7. The whole face was not covered.

The aim of these criteria is (a) to enable reliable coding, and (b) to include only photos where the depicted face-to-body ratio was an arbitrary decision of the author [11] and was not restricted by the presence of other “co-subjects” (e.g. a person depicted alongside a monument).

#### Inter-coder reliability

To measure inter-coder reliability of the face-ism index, an additional coder independently coded 60 randomly selected selfies (10 from each city). Calculated inter-coder reliability was high, *Krippendorff* α = .99.

The second coder also determined the gender of a depicted person in the same subset of selfies. There were no differences in determining the gender between the coders.

### Data analysis

To test differences in the face-ism index of selfies between men and women from all cities together and for each city separately, independent t-tests were conducted.

The *Shapiro-Wilk* test of normality, Q-Q plots and histograms were used to check for normality of the face-ism index data by city. *Levene’s* test was used to test homogeneity of variance between cities, and *Welch's* ANOVA was used to test the significance of mean differences. The *Games-Howell* post hoc procedure was used to determine which pairs of cities differed significantly. Significance was tested at the .05 level.

## Results

### Gender differences in face-ism index

The overall mean face-ism index is higher for men (*M* = .6214, *SD* = .19) than for women (*M* = .5767, *SD* = .18). The difference was significant *t*(1397.42) = 5.616, *p* < .001, *r* = .15, Cohen's *d* = .30. Levene's test indicated unequal variances (*F* = 11.32, *p* = .001).

### Cultural differences in face-ism

The face-ism index differed significantly between men and women in some cities, while the difference was not significant in other cities as presented in Table 1.

**Table 1:** Gender differences in face-ism index by city.

		Men		Women					
City	*N*	*N*	*M*	*N*	*M*	*t*	*df*	*p*	Cohen's *d*
(country)			*(SD)*		*(SD)*				
Bangkok	456	169	.6804	287	.6512	1.855	454	.064	.17412
(Thailand)			(.1661)		(.1545)				
Berlin	403	126	.6707	277	.6079	3.252	401	.001**	.32479
(Germany)			(.1765)		(.1812)				
London	408	130	.5790	278	.5306	2.317	406	.021*	.22998
(UK)			(.2075)		(.1914)				
Moscow	520	81	.5835	439	.5487	1.637	518	.102	.14385
(Russia)			(.1899)		(.1731)				
New York	417	136	.6208	281	.6058	0.719	228	.473	.09517
(USA)			(.2107)		(.1754)				
Sao Paolo	550	168	.5764	382	.5419	2.020	277	.044*	.24272
(Brazil)			(.5764)		(.5419)				

*p < .05.

** *p* < .01.

An analysis of variance (ANOVA) was used to examine differences in face-ism index scores between the cities included in this study. The *Shapiro-Wilk* test of normality indicated that the data at α < .05 level were not statistically normal. However, both the histograms and Normal *Q-Q plots* suggest approximately normal distributions for all cities. The *Levene's F* test indicated that homogeneity of variances was not met, *F*(5, 2748) = 4.47, *p* = .001. For this reason, the more robust *Welch’s F* test was used and yielded statistically significant differences in the face-ism index between the cities, *F*(5, 1250) = 36.57, *p* < 0.001, ω^2^ = .06. Owing to unequal variances, the *Games-Howell* post hoc procedure was used to determine which pairs of cities differed significantly. The results of post hoc analysis are presented in Table 2. The mean face-ism index and standard deviation are presented for each city and mean differences between cities were calculated. In cases where the mean difference is statistically significant, effect sizes in the form of Cohen’s d are presented in parentheses. Results show that a group of cities consisting of London, Sao Paulo and Moscow shows no statistically significant differences among city’s their mean face-ism indices. Also, two pairs of cities—Berlin and Bangkok and Berlin and New York—show no statistically significant differences in the face-ism index. A comparison of results for all other pairs of cities does yield statistically significant differences. Effect sizes indicate rather small difference between New York and other cities, with Cohen’s d ranging from .297 to .336. On the other hand, Bangkok differs the most from other cities, with Cohen’s d > .600 in most cases, and also indicating the highest mean value.

**Table 2:** Post hoc results for face-ism index differences by city.

	City	Mean	Mean differences (Cohen's d)					
		(SD)	1	2	3	4	5	6
1	Berlin	.628	—					
		(.182)						
2	Bangkok	.662	-.034	—				
		(.159)						
3	London	.546	.082*	.116*	—			
		(.198)	(.429)	(.648)				
4	Moscow	.554	.074*	.108*	-.008	—		
		(.176)	(.410)	(.654)				
5	Sao Paulo	.552	.076*	.110*	-.006	.002	—	
		(.174)	(.423)	(.660)				
6	New York	.611	.017	.051*	-.065*	-.057*	-.058*	—
		(.188)		(.297)	(.336)	(.311)	(.323)	

* The mean difference is significant at the 0.05 level.

### Socio-cultural correlates of face-ism

**Fig 1:**
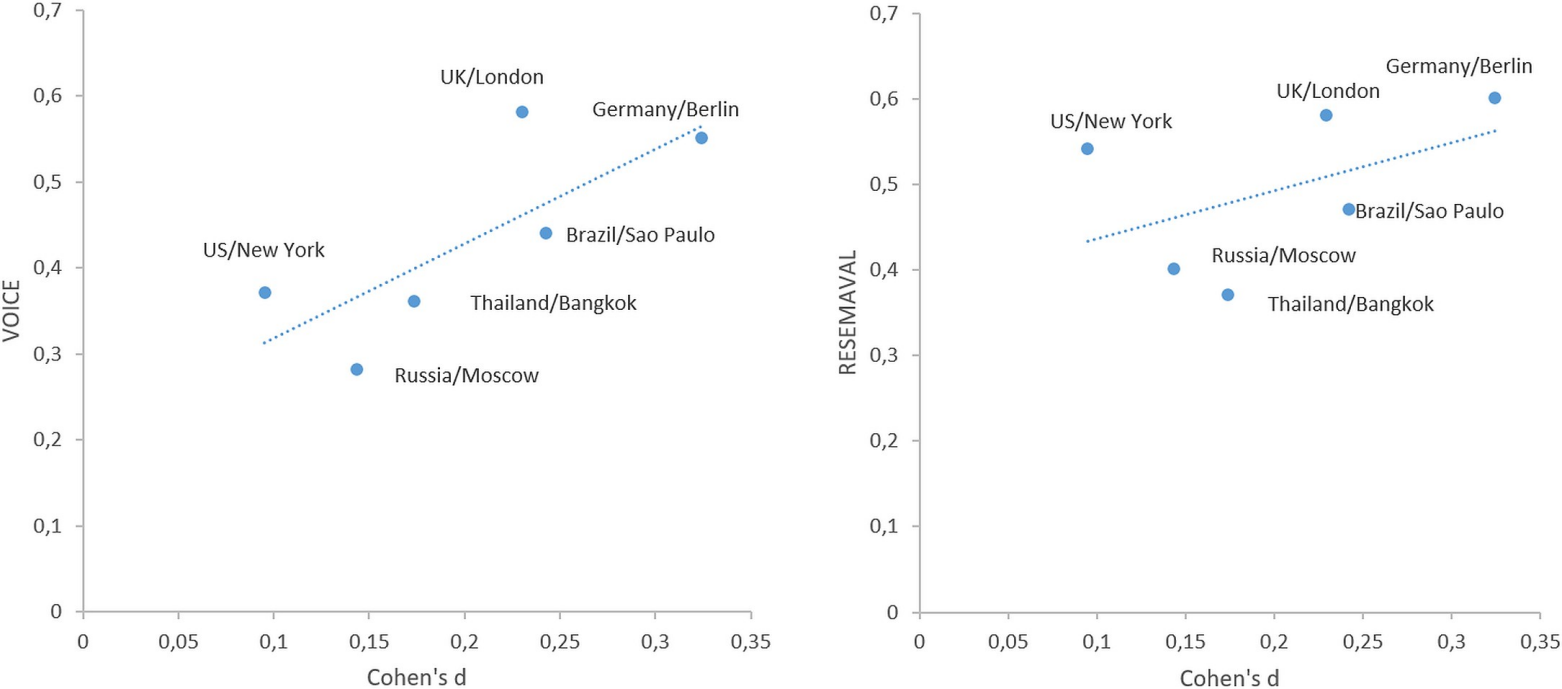
Scatter plots for face-ism Cohen’s d and WVS index RESEMAVAL and VOICE.

Next, we analysed the relation of face-ism to some potentially relevant measures of sociocultural context. Fig 1 presents scatter plots for Emancipative values (RESEMAVAL) as a general index and VOICE as a component of emancipative values. These are two indices that show a possible correlation with the expression of face-ism in selfies.

Correlations with face-ism did not reach a level of statistical significance and we present these results here as an indication pointing to possible further exploration. Spearman correlations of WVS and UNPD variables with face-ism Cohen’s d were calculated to identify possible candidates: SACSECVAL (r_s_ = -.03, p = .96), RESEMAVAL (r_s_ = .49, p = .33), AUTONOMY (r_s_ = .03, p = .96), EQUALITY (r_s_ = .03, p = .96), CHOICE (r_s_ = .29, p = .66), VOICE (r_s_ = .66, p = .16), HDI (r_s_ = .26, p = .62), GII (r_s_ = -.26, p = .62).

Correlations of the face-ism index measured as a mean value of facial prominence by city, and presented in Table 2, with sociocultural measures are SACSECVAL (r_s_ = 0.03, p = 0.96), RESEMAVAL (r_s_ = -0.26, p = 0.62), AUTONOMY (r_s_ = 0.20, p = 0.70), EQUALITY (r_s_ = -0.09, p = 0.87), CHOICE (r_s_ = -0.46, p = 0,35), VOICE (r_s_ = - 0.43, p = 0.40), HDI (r_s_ = -0.14, p = 0.79), GII (r_s_ = 0.029, p = 0.96).

## Discussion

Our results tend to support Archer’s initial findings that facial prominence of male faces can be found around the world and in different media. Even in selfies, where a person takes complete control over the image taking, editing and posting on public media, the phenomenon is still present. Hence, our first hypothesis is confirmed.

Facial prominence in male versus female selfies show no statistically significant differences in Moscow, Bangkok and especially New York; hence, our results do not statistically confirm Archer’s hypothesis on intercultural universality of the effect (H2), and therefore our H2 should be rejected. Of course, it does not mean that face-ism in those areas is non-existent. Nevertheless, it should be noted that the effect trends in the same direction as in the other three cities where face-ism was confirmed with statistically significant results. In all cases, it should be noted that the effect size is rather small.

When comparing our results with similar research on face-ism online, we found that image taking and posting behaviour in selfies shows results similar to those for self-selection of images for online profiles [18]. Also, control over the selection of images for professional use, as in the case of university professors and German politicians [19], retains gender-stereotypical differences. It seems that control over image taking, selection, editing and presentation does not mitigate the greater facial prominence of male faces in the images, and selfies are not an exception.

When comparing selfies’ face-ism with other characteristics of selfies, our results show that face-ism does not exaggerate gender-stereotypical representation, as has been found in other selfie content analysis. The analysis by Döring et al. [20] of Goffman’s gender display categories shows that selfies are more gender stereotypical in content compared to images in printed magazines.

One potential explanation is the complex nature of the selfie phenomenon. A difference in facial prominence is thus not related only to gender stereotypes. It is indeed present in contexts of gender inequality, researchers should also try to link the variation to cultural differences, even if the results are somewhat contradictory [25,31]. However, bias in facial prominence is also present in other contexts of inequality in social status, such as race and minority discrimination [9] or occupational status [14]. The stance toward the subject matter discussed by the particular medium is another source of diversity in facial prominence [15]. These influences could affect differences in facial prominence among groups and introduce some confusion in the understanding of the gender stereotypes present in face-ism. In our opinion, one more factor should be taken into consideration in relation to selfies, a factor which could influence the relative manifestation of face-ism. Because selfies are produced by the subject depicted in the image, the content composition is influenced by technology as well as by body limitations and the sensorimotor coordination skills of the author [32]. Therefore, even when selfies exaggerate gender stereotypical display in other aspects, such as Goffman’s gender display categories, face-ism would not necessarily resonate with those findings.

In seeking an explanation of why face-ism differs from country to country, we examined how effect sizes of face-ism resonate with well-established measures of sociocultural contexts. We explored several possibilities and compared face-ism effect sizes from the set of cities included in this research with HDI and GII from the UN Development Programme [29] and World Values Survey indices: SACSECVAL, RESEMAVAL and its components AUTONOMY, EQUALITY, CHOICE and VOICE [27]. Since the sample size of only 6 cities is very small, it was not possible to calculate statistically significant correlations; however, when comparing Pearson’s and Spearman’s correlation values, two indices stood out. It seems that the index reflecting emancipative values (RESEMAVAL) and especially its component VOICE do have some potential for explaining differences in facial prominence bias. In addition, the RESEMAVAL component CHOICE shows potential for future research. These findings should not be a surprise because emancipative values strengthen people’s desire for democracy, while enhancing human agency, and they are the single most important factor in advancing the empowerment of women [28]. However, GII from the UN Development Programme does not resonate with face-ism in selfies. It is a measure of gender inequality based on gender-based differences in life expectancy, education levels and economic empowerment. This resonates with WVS’s EQUALITY index, which indicates gender equality in jobs, politics and education and which also shows no correlation with face-ism.

In relation to the third hypothesis, our results show statistically significant differences among the cities, and post hoc analysis shows that some cities are more alike than others. Therefore, we can confirm that variation in face-ism index scores differs from country to country (H3). However, the explanation for this difference is not straightforward. When comparing our results to those of Smith and Cooley [18], for example New York and Sao Paulo display the results opposite to those from previous research. We explored several possibilities and compared the face-ism scores with HDI, GII, SACSECVAL, RESEMAVAL and its components. Our results do not indicate that mean face-ism index scores correlate with those measures. One exception might be the WVS’s CHOICE index (r_s_ = -.46, p = .35). This index reflects values marked by tolerance of homosexuality, abortion and divorce. It seems that people in more tolerant environments tend to express lower facial prominence. However, this question remains open for explanation and future research.

Besides some inconclusive results, another limitation of this study is related to the study sample. Even though there is no doubt that the sample is of a high quality and very systematically prepared [30], it has two constraints. The first problem is that only large and quite globalised cities are included. Further research should also involve non-urban populations and smaller cities, to gain a more balanced picture of specific countries included in the study. Another limitation, despite the fact that the cities were selected across the globe and cover a variety of cultural backgrounds, is that the data is available for only a few cities, one from each region. Future studies should include selfies from more cities and other countries, too.

The moderating role of the season/weather has not been taken into consideration in this research. Most of the cities included in the sample (New York, Berlin, Moscow and Bangkok) are located in the northern hemisphere; therefore, the photographs were taken in winter, during cold weather, while in Sao Paolo, which is in the southern hemisphere, the photographs were taken in summer, and during warm weather. The photographs in London were taken in the autumn. Involvement in various activities could influence the face-ism index; therefore, it is possible that seasonal conditions could influence the scores.

Selfies are used in online environments and thus also in intercultural communication settings. Therefore, future research should explore the influence of differences in facial prominence on impression formation across disparate cultures. While previous research revealed the consequences of facial prominence for different groups within one cultural setting, we hypothesise that differences in the face-ism index among several cultural backgrounds could potentially affect the perception of selfies from other cultures, even when there is no indication of previous bias based on stereotypes.

We can conclude that facial prominence bias is present in selfies, notwithstanding control by the subjects–the authors of the given self-portrait images. It is a complex phenomenon and is not related to gender stereotypes alone. For example, it remains an open issue whether sensorimotor coordination skills influence facial prominence work in the same direction as the reduction in gender inequality among participants from a specific cultural environment. Therefore, future research in face-ism theory should take into consideration the multifactor nature of facial prominence.

## Supporting information

S1 File Measurements of Bangkok selfies. (XLSX)

S2 File Measurements of Berlin selfies. (XLSX)

S3 File Measurements of London selfies. (XLSX)

S4 File Measurements of Moscow selfies. (XLSX)

S5 File Measurements of NewYork selfies. (XLSX)

S6 File Measurements of SaoPaulo selfies. (XLSX)
